# 5-[(*E*)-(2,6-Dichloro­benzyl­idene)amino]-2-hy­droxy­benzoic acid

**DOI:** 10.1107/S160053681004136X

**Published:** 2010-10-23

**Authors:** M. Nawaz Tahir, Hazoor Ahmad Shad, Muhammad Naeem Khan, Muhammad Ilyas Tariq

**Affiliations:** a Department of Physics, University of Sargodha, Sargodha, Pakistan; b Department of Chemistry, Govt. M. D. College, Toba Tek Singh, Punjab, Pakistan; c Applied Chemistry Research Center, PCSIR Laboratories Complex, Lahore 54600, Pakistan; d Department of Chemistry, University of Sargodha, Sargodha, Pakistan

## Abstract

There are two geometrically different mol­ecules in the asymmetric unit of the title compound, C_14_H_9_Cl_2_NO_3_. The 5-amino-2-hy­droxy­benzoic acid units [r.m.s. deviations of 0.0323 and 0.0414 Å] and 2,6-dichloro­benzaldehyde groups [r.m.s. deviations of 0.0285 and 0.0226 Å] are roughly planar and oriented at dihedral angles of 11.69 (13) and 83.12 (6)° in the two mol­ecules. An intra­molecular O—H⋯O hydrogen bond completes an *S*(6) ring motif in each mol­ecule. The two mol­ecules form dimers with each other through inter­molecular O—H⋯N and C—H⋯O hydrogen bonds, completing an *R*_2_^2^(8) ring motif. The dimers are inter­linked *via* inter­molecular O—H⋯N and C—H⋯O hydrogen bonds, forming polymeric sheets.

## Related literature

For a related structure, see: Tahir *et al.* (2010 ▶). For graph-set notation, see: Bernstein *et al.* (1995 ▶).
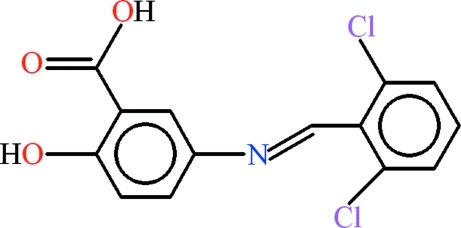


## Experimental

### 

#### Crystal data

C_14_H_9_Cl_2_NO_3_
*M* *_r_* = 310.12
Monoclinic, 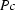

*a* = 10.4966 (10) Å
*b* = 4.8677 (4) Å
*c* = 26.300 (2) Å
β = 94.941 (4)°
*V* = 1338.8 (2) Å^3^
*Z* = 4
Mo *K*α radiation
μ = 0.49 mm^−1^
*T* = 296 K
0.28 × 0.18 × 0.15 mm

#### Data collection

Bruker Kappa APEXII CCD diffractometer
Absorption correction: multi-scan (*SADABS*; Bruker, 2005 ▶) *T*_min_ = 0.902, *T*_max_ = 0.928
21331 measured reflections
6487 independent reflections
3439 reflections with *I* > 2σ(*I*)
*R*_int_ = 0.068

#### Refinement

*R*[*F* ^2^ > 2σ(*F* ^2^)] = 0.059
*wR*(*F* ^2^) = 0.146
*S* = 1.02
6487 reflections
365 parameters
2 restraints
H-atom parameters constrained
Δρ_max_ = 0.34 e Å^−3^
Δρ_min_ = −0.30 e Å^−3^
Absolute structure: Flack (1983), 3138 Friedel pairs
Flack parameter: 0.17 (7)

### 

Data collection: *APEX2* (Bruker, 2009 ▶); cell refinement: *SAINT* (Bruker, 2009 ▶); data reduction: *SAINT*; program(s) used to solve structure: *SHELXS97* (Sheldrick, 2008 ▶); program(s) used to refine structure: *SHELXL97* (Sheldrick, 2008 ▶); molecular graphics: *ORTEP-3 for Windows* (Farrugia, 1997 ▶) and *PLATON* (Spek, 2009 ▶); software used to prepare material for publication: *WinGX* (Farrugia, 1999 ▶) and *PLATON*.

## Supplementary Material

Crystal structure: contains datablocks global, I. DOI: 10.1107/S160053681004136X/bq2240sup1.cif

Structure factors: contains datablocks I. DOI: 10.1107/S160053681004136X/bq2240Isup2.hkl

Additional supplementary materials:  crystallographic information; 3D view; checkCIF report

## Figures and Tables

**Table 1:** Hydrogen-bond geometry (Å, °)

*D*—H⋯*A*	*D*—H	H⋯*A*	*D*⋯*A*	*D*—H⋯*A*
O1—H1⋯N2	0.82	2.00	2.794 (5)	162
O3—H3⋯O2	0.82	1.87	2.585 (6)	146
O4—H4*A*⋯N1^i^	0.82	2.06	2.811 (5)	152
O6—H6⋯O5	0.82	1.87	2.586 (6)	145
C5—H5⋯O5^ii^	0.93	2.32	3.228 (6)	166
C26—H26⋯O2^iii^	0.93	2.45	3.267 (8)	147

Symmetry codes: (i) 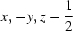; (ii) 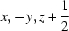; (iii) 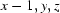.

**Fig. 1.**
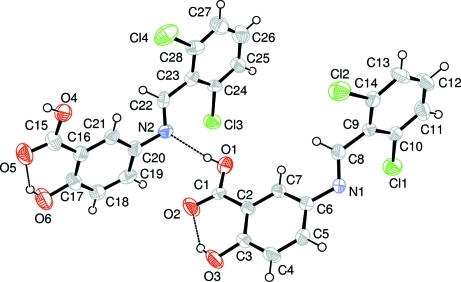
View of the title compound with the atom numbering scheme. The thermal ellipsoids are drawn at the 50% probability level. The dotted lines represent the inter and intramolecular H-bondings.

**Fig. 2.**
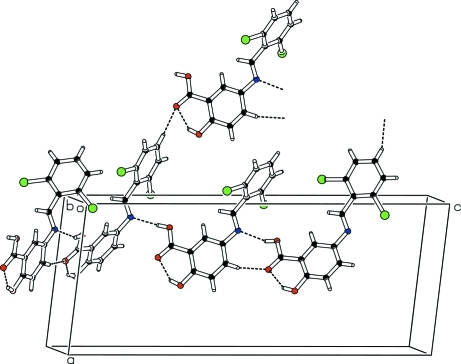
The partial packing (PLATON; Spek, 2009) which shows that molecules form dimers which are interlinked and form polymeric sheets.

## supplementary crystallographic information

### Comment

The title compound (I, Fig. 1) is being reported as a part of our on going project related to synthesize various Schiff bases of 5-amino-2-hydroxybenzoic acid and 2,6-dichlorobenzaldehyde with different aldehydes and anilines, respectively. In this context, we have recently reported the synthesis and crystal structure of 2-hydroxy-5-{[(*E*)-4-methoxybenzylidene]azaniumyl} benzoate (Tahir *et al.*, 2010).

The title compound consists of two molecules in the crystallographic asymmetric unit which differ from each other geometrically. In one molecule, the 5-amino-2-hydroxybenzoic acid moiety A (C1—C7/N1/O1—O3) and group B (C8—C14/CL1/CL2) of 2,6-dichlorobenzaldehyde are planar with r. m. s deviation of 0.0414 and 0.0226 Å, respectively. The dihedral angle between A/B is 83.12 (6)°. In second molecule, the 5-amino-2-hydroxybenzoic acid moiety C (C15—C21/N2/O4—O6) and group D (C22—C28/CL3/CL4) of 2,6-dichlorobenzaldehyde are also planar with r. m. s deviation of 0.0323 and 0.0285 Å, respectively. The dihedral angle between C/D is 11.69 (13)°. In each molecule there exist an S(6) ring motif (Bernstein *et al.*, 1995) due to intramolecular H-bonding of O—H···O type (Table 1, Fig 1). The molecules are stabilized in the form of dimers due to O—H···N and C—H···O types of intermolecular H-bondings with *R*_2_^2^(8) ring motifs (Table 1, Fig. 2). The dimers are interlinked due to O—H···N and C—H···O type of intermolecular H-bondings to form polymeric sheets (Table 1, Fig. 2).

### Experimental

Equimolar quantities of 5-amino-2-hydroxybenzoic acid and 2,6-dichlorobenzaldehyde were refluxed in methanol for 30 min resulting in orange yellow solution. The solution was kept at room temperature which afforded colorless prisms after a week.

### Refinement

The H-atoms were positioned geometrically (O–H = 0.82, C–H = 0.93 Å) and were included in the refinement in the riding model approximation, with *U*_iso_(H) = *xU*_eq_(C, O), where *x* = 1.2 for all H-atoms.

### Crystal data

**Table:**

C_14_H_9_Cl_2_NO_3_	*F*(000) = 632
*M**_r_* = 310.12	*D*_x_ = 1.539 Mg m^−^^3^
Monoclinic, *P**c*	Mo *K*α radiation, λ = 0.71073 Å
Hall symbol: P -2yc	Cell parameters from 3439 reflections
*a* = 10.4966 (10) Å	θ = 1.6–28.3°
*b* = 4.8677 (4) Å	µ = 0.49 mm^−^^1^
*c* = 26.300 (2) Å	*T* = 296 K
β = 94.941 (4)°	Prism, colorless
*V* = 1338.8 (2) Å^3^	0.28 × 0.18 × 0.15 mm
*Z* = 4	

### Data collection

**Table:**

Bruker Kappa APEXII CCD diffractometer	6487 independent reflections
Radiation source: fine-focus sealed tube	3439 reflections with *I* > 2σ(*I*)
graphite	*R*_int_ = 0.068
Detector resolution: 7.50 pixels mm^-1^	θ_max_ = 28.3°, θ_min_ = 1.6°
ω scans	*h* = −13→13
Absorption correction: multi-scan (*SADABS*; Bruker, 2005)	*k* = −5→6
*T*_min_ = 0.902, *T*_max_ = 0.928	*l* = −35→35
21331 measured reflections	

### Refinement

**Table:**

Refinement on *F*^2^	Secondary atom site location: difference Fourier map
Least-squares matrix: full	Hydrogen site location: inferred from neighbouring sites
*R*[*F*^2^ > 2σ(*F*^2^)] = 0.059	H-atom parameters constrained
*wR*(*F*^2^) = 0.146	*w* = 1/[σ^2^(*F*_o_^2^) + (0.0569*P*)^2^] where *P* = (*F*_o_^2^ + 2*F*_c_^2^)/3
*S* = 1.02	(Δ/σ)_max_ < 0.001
6487 reflections	Δρ_max_ = 0.34 e Å^−^^3^
365 parameters	Δρ_min_ = −0.29 e Å^−^^3^
2 restraints	Absolute structure: Flack (1983), 3138 Friedel pairs
Primary atom site location: structure-invariant direct methods	Flack parameter: 0.17 (7)

### Special details

**Table:**

Geometry. Bond distances, angles *etc*. have been calculated using the rounded fractional coordinates. All su's are estimated from the variances of the (full) variance-covariance matrix. The cell e.s.d.'s are taken into account in the estimation of distances, angles and torsion angles
Refinement. Refinement of *F*^2^ against ALL reflections. The weighted *R*-factor *wR* and goodness of fit *S* are based on *F*^2^, conventional *R*-factors *R* are based on *F*, with *F* set to zero for negative *F*^2^. The threshold expression of *F*^2^ > σ(*F*^2^) is used only for calculating *R*-factors(gt) *etc*. and is not relevant to the choice of reflections for refinement. *R*-factors based on *F*^2^ are statistically about twice as large as those based on *F*, and *R*- factors based on ALL data will be even larger.

### Fractional atomic coordinates and isotropic or equivalent isotropic displacement parameters (Å^2^)

**Table:**

	*x*	*y*	*z*	*U*_iso_*/*U*_eq_	
Cl1	0.01640 (13)	0.2386 (3)	0.50290 (5)	0.0558 (5)	
Cl2	−0.11347 (14)	−0.4868 (3)	0.35136 (6)	0.0612 (5)	
O1	0.1945 (3)	0.6393 (7)	0.27742 (13)	0.0492 (12)	
O2	0.3893 (4)	0.6063 (10)	0.25333 (16)	0.0790 (18)	
O3	0.5582 (3)	0.3127 (9)	0.30599 (17)	0.0702 (16)	
N1	0.1729 (3)	−0.0404 (8)	0.42295 (14)	0.0379 (12)	
C1	0.3099 (5)	0.5406 (10)	0.28246 (19)	0.0411 (17)	
C2	0.3380 (4)	0.3473 (9)	0.32461 (17)	0.0347 (17)	
C3	0.4612 (4)	0.2384 (10)	0.33363 (18)	0.0415 (17)	
C4	0.4876 (5)	0.0445 (11)	0.3719 (2)	0.0521 (19)	
C5	0.3935 (5)	−0.0406 (11)	0.40083 (19)	0.0467 (17)	
C6	0.2681 (4)	0.0599 (10)	0.39217 (17)	0.0374 (17)	
C7	0.2438 (4)	0.2548 (9)	0.35459 (17)	0.0360 (17)	
C8	0.0585 (4)	−0.0667 (9)	0.40207 (18)	0.0383 (17)	
C9	−0.0529 (4)	−0.1379 (9)	0.42924 (18)	0.0339 (16)	
C10	−0.0825 (5)	−0.0117 (10)	0.47478 (18)	0.0378 (17)	
C11	−0.1935 (5)	−0.0696 (11)	0.4974 (2)	0.052 (2)	
C12	−0.2784 (5)	−0.2615 (12)	0.4752 (2)	0.056 (2)	
C13	−0.2541 (5)	−0.3906 (11)	0.4302 (2)	0.0523 (19)	
C14	−0.1424 (4)	−0.3278 (10)	0.40825 (17)	0.0400 (17)	
Cl3	0.00607 (11)	1.1937 (3)	0.24864 (5)	0.0512 (5)	
Cl4	−0.18665 (17)	0.4321 (3)	0.11234 (6)	0.0776 (7)	
O4	0.2281 (3)	0.3028 (7)	0.01751 (13)	0.0463 (12)	
O5	0.4067 (4)	0.4878 (8)	−0.00825 (15)	0.0631 (17)	
O6	0.5271 (4)	0.8590 (10)	0.04744 (16)	0.0689 (17)	
N2	0.1341 (4)	0.8088 (8)	0.17673 (14)	0.0382 (12)	
C15	0.3276 (5)	0.4741 (10)	0.0230 (2)	0.0451 (19)	
C16	0.3311 (4)	0.6457 (9)	0.06853 (17)	0.0370 (17)	
C17	0.4292 (5)	0.8315 (10)	0.0784 (2)	0.0444 (17)	
C18	0.4326 (5)	1.0003 (10)	0.12045 (19)	0.0458 (17)	
C19	0.3375 (5)	0.9882 (10)	0.1538 (2)	0.0475 (17)	
C20	0.2365 (5)	0.8112 (10)	0.14381 (18)	0.0402 (17)	
C21	0.2333 (5)	0.6344 (10)	0.10117 (18)	0.0390 (17)	
C22	0.0207 (5)	0.8039 (10)	0.15470 (18)	0.0390 (17)	
C23	−0.0965 (5)	0.8056 (10)	0.18221 (18)	0.0382 (16)	
C24	−0.1139 (4)	0.9731 (10)	0.22433 (18)	0.0390 (17)	
C25	−0.2274 (5)	0.9847 (11)	0.2464 (2)	0.0475 (19)	
C26	−0.3300 (6)	0.8264 (12)	0.2267 (2)	0.063 (2)	
C27	−0.3158 (6)	0.6557 (12)	0.1858 (2)	0.064 (2)	
C28	−0.1997 (5)	0.6470 (10)	0.1647 (2)	0.0489 (19)	
H1	0.18323	0.72156	0.25020	0.0590*	
H3	0.53240	0.42574	0.28439	0.0842*	
H4	0.56965	−0.02728	0.37779	0.0623*	
H5	0.41260	−0.16763	0.42678	0.0556*	
H7	0.16174	0.32691	0.34908	0.0432*	
H8	0.04532	−0.03843	0.36704	0.0458*	
H11	−0.21103	0.01950	0.52729	0.0619*	
H12	−0.35230	−0.30385	0.49065	0.0671*	
H13	−0.31173	−0.51732	0.41495	0.0627*	
H4A	0.23581	0.20059	−0.00682	0.0555*	
H6	0.51301	0.76141	0.02216	0.0830*	
H18	0.49964	1.12414	0.12658	0.0550*	
H19	0.34199	1.09900	0.18268	0.0571*	
H21	0.16627	0.51077	0.09487	0.0465*	
H22	0.01127	0.79900	0.11922	0.0470*	
H25	−0.23562	1.09813	0.27436	0.0572*	
H26	−0.40778	0.83527	0.24111	0.0755*	
H27	−0.38370	0.54748	0.17242	0.0765*	

### Atomic displacement parameters (Å^2^)

**Table:**

	*U*^11^	*U*^22^	*U*^33^	*U*^12^	*U*^13^	*U*^23^
Cl1	0.0538 (9)	0.0637 (9)	0.0493 (8)	−0.0071 (7)	0.0009 (7)	−0.0136 (6)
Cl2	0.0558 (9)	0.0692 (9)	0.0576 (10)	−0.0025 (7)	−0.0003 (7)	−0.0188 (7)
O1	0.053 (2)	0.053 (2)	0.041 (2)	0.0001 (17)	0.0012 (17)	0.0127 (16)
O2	0.043 (2)	0.118 (4)	0.078 (3)	−0.002 (2)	0.017 (2)	0.059 (3)
O3	0.035 (2)	0.102 (3)	0.077 (3)	0.011 (2)	0.024 (2)	0.037 (2)
N1	0.033 (2)	0.053 (2)	0.028 (2)	−0.0026 (18)	0.0037 (19)	0.0082 (18)
C1	0.028 (3)	0.061 (3)	0.033 (3)	−0.004 (2)	−0.005 (2)	0.011 (2)
C2	0.029 (3)	0.037 (3)	0.038 (3)	−0.0014 (19)	0.003 (2)	0.004 (2)
C3	0.030 (3)	0.053 (3)	0.042 (3)	−0.005 (2)	0.006 (2)	0.008 (2)
C4	0.029 (3)	0.063 (3)	0.065 (4)	0.009 (2)	0.008 (3)	0.021 (3)
C5	0.043 (3)	0.059 (3)	0.038 (3)	0.004 (2)	0.003 (3)	0.018 (2)
C6	0.033 (3)	0.045 (3)	0.034 (3)	−0.005 (2)	0.002 (2)	0.002 (2)
C7	0.030 (3)	0.045 (3)	0.033 (3)	−0.004 (2)	0.003 (2)	−0.003 (2)
C8	0.041 (3)	0.043 (3)	0.031 (3)	−0.006 (2)	0.004 (2)	0.001 (2)
C9	0.027 (3)	0.041 (2)	0.033 (3)	−0.002 (2)	−0.001 (2)	0.007 (2)
C10	0.033 (3)	0.046 (3)	0.034 (3)	0.002 (2)	0.001 (2)	0.010 (2)
C11	0.051 (4)	0.057 (3)	0.049 (4)	0.007 (3)	0.018 (3)	0.008 (3)
C12	0.031 (3)	0.074 (4)	0.066 (4)	−0.001 (3)	0.015 (3)	0.015 (3)
C13	0.030 (3)	0.055 (3)	0.072 (4)	−0.007 (2)	0.005 (3)	0.009 (3)
C14	0.041 (3)	0.047 (3)	0.032 (3)	0.002 (2)	0.003 (2)	0.002 (2)
Cl3	0.0422 (8)	0.0615 (8)	0.0498 (8)	−0.0071 (6)	0.0038 (6)	−0.0177 (7)
Cl4	0.0938 (13)	0.0711 (10)	0.0639 (11)	−0.0065 (9)	−0.0170 (9)	−0.0238 (9)
O4	0.047 (2)	0.051 (2)	0.041 (2)	0.0006 (17)	0.0052 (17)	−0.0137 (16)
O5	0.062 (3)	0.077 (3)	0.054 (3)	−0.009 (2)	0.026 (2)	−0.022 (2)
O6	0.063 (3)	0.082 (3)	0.066 (3)	−0.019 (2)	0.030 (2)	−0.016 (2)
N2	0.031 (2)	0.049 (2)	0.034 (2)	0.0057 (18)	−0.001 (2)	−0.0005 (18)
C15	0.049 (4)	0.037 (3)	0.051 (3)	0.005 (2)	0.015 (3)	−0.006 (2)
C16	0.039 (3)	0.036 (3)	0.036 (3)	0.008 (2)	0.004 (2)	0.001 (2)
C17	0.042 (3)	0.043 (3)	0.050 (3)	0.006 (2)	0.014 (3)	0.002 (2)
C18	0.043 (3)	0.052 (3)	0.042 (3)	−0.009 (2)	0.001 (3)	−0.007 (2)
C19	0.045 (3)	0.051 (3)	0.044 (3)	−0.003 (2)	−0.010 (3)	−0.008 (2)
C20	0.041 (3)	0.047 (3)	0.033 (3)	0.007 (2)	0.005 (2)	0.000 (2)
C21	0.041 (3)	0.040 (3)	0.035 (3)	0.005 (2)	−0.002 (2)	0.006 (2)
C22	0.048 (3)	0.044 (3)	0.026 (3)	0.004 (2)	0.009 (2)	0.002 (2)
C23	0.045 (3)	0.037 (2)	0.032 (3)	0.005 (2)	0.000 (2)	0.000 (2)
C24	0.030 (3)	0.047 (3)	0.039 (3)	−0.006 (2)	−0.003 (2)	0.003 (2)
C25	0.038 (3)	0.057 (3)	0.048 (4)	0.001 (2)	0.007 (3)	0.006 (2)
C26	0.057 (4)	0.067 (4)	0.064 (4)	−0.005 (3)	0.005 (3)	0.015 (3)
C27	0.063 (4)	0.059 (4)	0.069 (4)	−0.026 (3)	0.006 (3)	0.003 (3)
C28	0.045 (3)	0.045 (3)	0.055 (4)	−0.004 (2)	−0.006 (3)	−0.006 (2)

### Geometric parameters (Å, °)

**Table:**

Cl1—C10	1.725 (5)	C12—C13	1.383 (8)
Cl2—C14	1.734 (5)	C13—C14	1.385 (7)
Cl3—C24	1.735 (5)	C4—H4	0.9300
Cl4—C28	1.744 (5)	C5—H5	0.9300
O1—C1	1.299 (6)	C7—H7	0.9300
O2—C1	1.222 (7)	C8—H8	0.9300
O3—C3	1.350 (6)	C11—H11	0.9300
O1—H1	0.8200	C12—H12	0.9300
O3—H3	0.8200	C13—H13	0.9300
O4—C15	1.335 (6)	C15—C16	1.458 (7)
O5—C15	1.219 (7)	C16—C21	1.395 (7)
O6—C17	1.372 (7)	C16—C17	1.378 (7)
O4—H4A	0.8200	C17—C18	1.376 (7)
O6—H6	0.8200	C18—C19	1.386 (7)
N1—C6	1.425 (6)	C19—C20	1.374 (7)
N1—C8	1.283 (5)	C20—C21	1.412 (7)
N2—C22	1.278 (7)	C22—C23	1.480 (7)
N2—C20	1.437 (6)	C23—C28	1.377 (7)
C1—C2	1.465 (7)	C23—C24	1.400 (7)
C2—C7	1.392 (6)	C24—C25	1.371 (7)
C2—C3	1.399 (6)	C25—C26	1.388 (8)
C3—C4	1.390 (7)	C26—C27	1.377 (8)
C4—C5	1.362 (7)	C27—C28	1.383 (8)
C5—C6	1.404 (7)	C18—H18	0.9300
C6—C7	1.378 (6)	C19—H19	0.9300
C8—C9	1.464 (6)	C21—H21	0.9300
C9—C14	1.397 (6)	C22—H22	0.9300
C9—C10	1.405 (7)	C25—H25	0.9300
C10—C11	1.382 (7)	C26—H26	0.9300
C11—C12	1.385 (8)	C27—H27	0.9300
			
Cl1···N1	3.092 (4)	C16···C18^iv^	3.552 (7)
Cl1···C9^i^	3.640 (5)	C16···Cl1^x^	3.637 (4)
Cl1···C14^i^	3.566 (5)	C17···C15^i^	3.575 (7)
Cl1···O4^ii^	3.447 (4)	C18···C27^viii^	3.458 (8)
Cl1···O4^iii^	3.150 (4)	C18···C15^i^	3.552 (7)
Cl1···C15^iii^	3.551 (5)	C18···C16^i^	3.552 (7)
Cl1···C16^iii^	3.637 (5)	C19···C21^i^	3.570 (7)
Cl1···C21^iii^	3.351 (5)	C19···O2	3.218 (7)
Cl2···C8^iv^	3.548 (5)	C20···O2	3.327 (6)
Cl2···Cl3^v^	3.446 (2)	C21···C19^iv^	3.570 (7)
Cl3···O1	3.393 (4)	C21···Cl1^x^	3.351 (5)
Cl3···C23^i^	3.572 (5)	C23···Cl3^iv^	3.572 (5)
Cl3···C7^i^	3.589 (5)	C23···Cl4^i^	3.643 (5)
Cl3···O1^i^	2.989 (4)	C25···O3^xiii^	3.268 (7)
Cl3···N2	3.055 (4)	C26···O2^xiv^	3.267 (8)
Cl3···Cl2^vi^	3.446 (2)	C27···C18^xiv^	3.458 (8)
Cl4···C11^vii^	3.496 (5)	C1···H3	2.4000
Cl4···C23^iv^	3.643 (5)	C6···H4A^ii^	2.9900
Cl1···H22^iii^	3.0700	C7···H8	2.5700
Cl1···H21^iii^	3.0300	C8···H7	2.6500
Cl2···H8	2.7600	C8···H4A^ii^	2.9800
Cl2···H25^v^	3.0600	C13···H4^xiv^	2.8300
Cl2···H7^iv^	3.0300	C15···H6	2.4000
Cl3···H1	2.9500	C20···H1	2.9300
Cl3···H7^i^	3.0500	C21···H22	2.5500
Cl4···H22	2.7300	C22···H21	2.6900
Cl4···H11^vii^	3.1300	C22···H1	2.9400
O1···Cl3	3.393 (4)	C26···H3^xiv^	2.9300
O1···N2	2.794 (5)	H1···Cl3	2.9500
O1···Cl3^iv^	2.989 (4)	H1···N2	2.0000
O2···C26^viii^	3.267 (8)	H1···C20	2.9300
O2···O3	2.585 (6)	H1···C22	2.9400
O2···C19	3.218 (7)	H3···H26^viii^	2.4100
O2···C20	3.327 (6)	H3···C26^viii^	2.9300
O3···C25^ix^	3.268 (7)	H3···O2	1.8700
O3···O2	2.585 (6)	H3···C1	2.4000
O4···Cl1^vii^	3.447 (4)	H4···C13^viii^	2.8300
O4···N1^vii^	2.811 (5)	H4A···C8^vii^	2.9800
O4···Cl1^x^	3.150 (4)	H4A···N1^vii^	2.0600
O5···O6	2.586 (6)	H4A···C6^vii^	2.9900
O5···C5^vii^	3.228 (6)	H5···O5^ii^	2.3200
O6···O5	2.586 (6)	H6···C15	2.4000
O1···H7	2.4700	H6···O5	1.8700
O2···H3	1.8700	H7···Cl2^i^	3.0300
O2···H26^viii^	2.4500	H7···H8	2.2300
O3···H25^ix^	2.6000	H7···C8	2.6500
O4···H21	2.4100	H7···O1	2.4700
O5···H6	1.8700	H7···Cl3^iv^	3.0500
O5···H5^vii^	2.3200	H8···H7	2.2300
O5···H12^xi^	2.6900	H8···C7	2.5700
O6···H11^xii^	2.9000	H8···Cl2	2.7600
N1···Cl1	3.092 (4)	H11···Cl4^ii^	3.1300
N1···O4^ii^	2.811 (5)	H11···O6^xv^	2.9000
N2···Cl3	3.055 (4)	H12···O5^xvi^	2.6900
N2···O1	2.794 (5)	H21···O4	2.4100
N1···H4A^ii^	2.0600	H21···C22	2.6900
N2···H1	2.0000	H21···H22	2.2800
C5···O5^ii^	3.228 (6)	H21···Cl1^x^	3.0300
C7···Cl3^iv^	3.589 (5)	H22···Cl4	2.7300
C8···Cl2^i^	3.548 (5)	H22···C21	2.5500
C9···Cl1^iv^	3.640 (5)	H22···H21	2.2800
C11···Cl4^ii^	3.496 (6)	H22···Cl1^x^	3.0700
C14···Cl1^iv^	3.566 (5)	H25···Cl2^vi^	3.0600
C15···Cl1^x^	3.551 (5)	H25···O3^xiii^	2.6000
C15···C18^iv^	3.552 (7)	H26···O2^xiv^	2.4500
C15···C17^iv^	3.575 (7)	H26···H3^xiv^	2.4100
			
C1—O1—H1	109.00	C13—C12—H12	120.00
C3—O3—H3	109.00	C14—C13—H13	121.00
C15—O4—H4A	110.00	C12—C13—H13	121.00
C17—O6—H6	109.00	O4—C15—C16	114.3 (4)
C6—N1—C8	117.9 (4)	O5—C15—C16	123.5 (5)
C20—N2—C22	116.3 (4)	O4—C15—O5	122.2 (5)
O2—C1—C2	122.6 (5)	C17—C16—C21	119.2 (4)
O1—C1—O2	121.3 (5)	C15—C16—C17	119.8 (4)
O1—C1—C2	116.1 (4)	C15—C16—C21	121.0 (4)
C3—C2—C7	118.2 (4)	C16—C17—C18	120.7 (5)
C1—C2—C3	119.7 (4)	O6—C17—C16	122.6 (5)
C1—C2—C7	122.0 (4)	O6—C17—C18	116.8 (5)
O3—C3—C4	117.3 (4)	C17—C18—C19	120.9 (5)
O3—C3—C2	122.4 (4)	C18—C19—C20	119.5 (5)
C2—C3—C4	120.3 (4)	N2—C20—C21	120.4 (4)
C3—C4—C5	120.1 (5)	N2—C20—C19	119.7 (4)
C4—C5—C6	121.2 (5)	C19—C20—C21	120.0 (5)
N1—C6—C5	118.8 (4)	C16—C21—C20	119.8 (5)
N1—C6—C7	123.2 (4)	N2—C22—C23	124.0 (4)
C5—C6—C7	118.0 (4)	C22—C23—C28	119.8 (4)
C2—C7—C6	122.2 (4)	C22—C23—C24	124.1 (4)
N1—C8—C9	124.9 (4)	C24—C23—C28	116.0 (5)
C10—C9—C14	115.8 (4)	Cl3—C24—C25	116.6 (4)
C8—C9—C14	120.1 (4)	Cl3—C24—C23	120.8 (4)
C8—C9—C10	123.9 (4)	C23—C24—C25	122.4 (4)
Cl1—C10—C9	120.4 (4)	C24—C25—C26	119.6 (5)
Cl1—C10—C11	117.3 (4)	C25—C26—C27	119.6 (6)
C9—C10—C11	122.3 (5)	C26—C27—C28	119.4 (5)
C10—C11—C12	119.5 (5)	C23—C28—C27	123.0 (5)
C11—C12—C13	120.5 (5)	Cl4—C28—C23	119.2 (4)
C12—C13—C14	118.8 (5)	Cl4—C28—C27	117.9 (4)
Cl2—C14—C9	118.3 (3)	C17—C18—H18	120.00
Cl2—C14—C13	118.6 (4)	C19—C18—H18	120.00
C9—C14—C13	123.1 (4)	C18—C19—H19	120.00
C5—C4—H4	120.00	C20—C19—H19	120.00
C3—C4—H4	120.00	C16—C21—H21	120.00
C4—C5—H5	119.00	C20—C21—H21	120.00
C6—C5—H5	119.00	N2—C22—H22	118.00
C2—C7—H7	119.00	C23—C22—H22	118.00
C6—C7—H7	119.00	C24—C25—H25	120.00
C9—C8—H8	118.00	C26—C25—H25	120.00
N1—C8—H8	118.00	C25—C26—H26	120.00
C10—C11—H11	120.00	C27—C26—H26	120.00
C12—C11—H11	120.00	C26—C27—H27	120.00
C11—C12—H12	120.00	C28—C27—H27	120.00
			
C8—N1—C6—C5	−145.6 (5)	C11—C12—C13—C14	1.1 (8)
C8—N1—C6—C7	35.4 (7)	C12—C13—C14—C9	−0.5 (8)
C6—N1—C8—C9	−173.1 (4)	C12—C13—C14—Cl2	−179.1 (4)
C20—N2—C22—C23	−178.9 (4)	O5—C15—C16—C17	−1.4 (8)
C22—N2—C20—C21	−44.4 (6)	O5—C15—C16—C21	175.6 (5)
C22—N2—C20—C19	135.2 (5)	O4—C15—C16—C17	−179.9 (4)
O2—C1—C2—C7	−174.0 (5)	O4—C15—C16—C21	−3.0 (7)
O1—C1—C2—C7	5.8 (7)	C21—C16—C17—C18	1.4 (7)
O1—C1—C2—C3	−178.0 (4)	C15—C16—C17—C18	178.3 (5)
O2—C1—C2—C3	2.3 (7)	C21—C16—C17—O6	−178.5 (5)
C7—C2—C3—C4	−0.5 (7)	C17—C16—C21—C20	−0.4 (7)
C7—C2—C3—O3	179.0 (4)	C15—C16—C17—O6	−1.5 (7)
C1—C2—C3—O3	2.6 (7)	C15—C16—C21—C20	−177.3 (4)
C3—C2—C7—C6	−0.5 (7)	C16—C17—C18—C19	−0.2 (8)
C1—C2—C7—C6	175.8 (4)	O6—C17—C18—C19	179.7 (5)
C1—C2—C3—C4	−176.8 (5)	C17—C18—C19—C20	−2.0 (8)
C2—C3—C4—C5	0.0 (8)	C18—C19—C20—C21	3.0 (7)
O3—C3—C4—C5	−179.5 (5)	C18—C19—C20—N2	−176.6 (4)
C3—C4—C5—C6	1.3 (8)	N2—C20—C21—C16	177.8 (4)
C4—C5—C6—C7	−2.2 (7)	C19—C20—C21—C16	−1.8 (7)
C4—C5—C6—N1	178.8 (5)	N2—C22—C23—C24	42.9 (7)
C5—C6—C7—C2	1.8 (7)	N2—C22—C23—C28	−141.0 (5)
N1—C6—C7—C2	−179.2 (4)	C22—C23—C24—Cl3	−1.1 (7)
N1—C8—C9—C14	−137.2 (5)	C22—C23—C24—C25	174.8 (5)
N1—C8—C9—C10	47.5 (7)	C28—C23—C24—Cl3	−177.4 (4)
C14—C9—C10—C11	−0.4 (7)	C28—C23—C24—C25	−1.4 (7)
C8—C9—C10—C11	175.1 (5)	C22—C23—C28—Cl4	3.6 (7)
C8—C9—C14—C13	−175.5 (5)	C22—C23—C28—C27	−174.4 (5)
C10—C9—C14—Cl2	178.8 (4)	C24—C23—C28—Cl4	180.0 (4)
C8—C9—C14—Cl2	3.1 (6)	C24—C23—C28—C27	2.0 (8)
C8—C9—C10—Cl1	−1.4 (7)	Cl3—C24—C25—C26	176.0 (4)
C14—C9—C10—Cl1	−176.9 (4)	C23—C24—C25—C26	−0.2 (8)
C10—C9—C14—C13	0.1 (7)	C24—C25—C26—C27	1.2 (8)
C9—C10—C11—C12	1.0 (8)	C25—C26—C27—C28	−0.6 (8)
Cl1—C10—C11—C12	177.6 (4)	C26—C27—C28—Cl4	−179.0 (4)
C10—C11—C12—C13	−1.3 (8)	C26—C27—C28—C23	−1.0 (8)

Symmetry codes: (i) *x*, *y*+1, *z*; (ii) *x*, −*y*, *z*+1/2; (iii) *x*, −*y*+1, *z*+1/2; (iv) *x*, *y*−1, *z*; (v) *x*, *y*−2, *z*; (vi) *x*, *y*+2, *z*; (vii) *x*, −*y*, *z*−1/2; (viii) *x*+1, *y*, *z*; (ix) *x*+1, *y*−1, *z*; (x) *x*, −*y*+1, *z*−1/2; (xi) *x*+1, −*y*, *z*−1/2; (xii) *x*+1, −*y*+1, *z*−1/2; (xiii) *x*−1, *y*+1, *z*; (xiv) *x*−1, *y*, *z*; (xv) *x*−1, −*y*+1, *z*+1/2; (xvi) *x*−1, −*y*, *z*+1/2.

### Hydrogen-bond geometry (Å, °)

**Table:**

*D*—H···*A*	*D*—H	H···*A*	*D*···*A*	*D*—H···*A*
O1—H1···N2	0.82	2.00	2.794 (5)	162
O3—H3···O2	0.82	1.87	2.585 (6)	146
O4—H4A···N1^vii^	0.82	2.06	2.811 (5)	152
O6—H6···O5	0.82	1.87	2.586 (6)	145
C5—H5···O5^ii^	0.93	2.32	3.228 (6)	166
C26—H26···O2^xiv^	0.93	2.45	3.267 (8)	147

Symmetry codes: (vii) *x*, −*y*, *z*−1/2; (ii) *x*, −*y*, *z*+1/2; (xiv) *x*−1, *y*, *z*.
